# Obesity and metabolic syndrome in adults with a 22q11.2 microdeletion

**DOI:** 10.1038/s41366-024-01685-2

**Published:** 2024-11-30

**Authors:** Hester Jaspers Faijer-Westerink, Emma N. M. M. von Scheibler, Elisabeth F. C. van Rossum, Mieke M. van Haelst, Claudia Vingerhoets, Thérèse A. M. J. van Amelsvoort, Agnies M. van Eeghen, Erik Boot

**Affiliations:** 1 Advisium, ‘s Heeren Loo Zorggroep, Amersfoort, The Netherlands; 2 Koraal, Maastricht, The Netherlands; 3 Department of Psychiatry and Neuropsychology, MHeNs, Maastricht University, Maastricht, The Netherlands; 4 Department of Internal Medicine, Division of Endocrinology, Erasmus MC, University Medical Center Rotterdam, Rotterdam, The Netherlands; 5 Obesity Center CGG, Erasmus MC, University Medical Center Rotterdam, Rotterdam, The Netherlands; 6 Department of Human Genetics, Amsterdam Reproduction & Development Research Institute, Amsterdam UMC, University of Amsterdam, Amsterdam, The Netherlands; 7 Emma Center for Personalized Medicine, Amsterdam UMC, University of Amsterdam, Amsterdam, The Netherlands; 8 Emma Children’s Hospital, University of Amsterdam, Amsterdam, The Netherlands; 9 The Dalglish Family 22q Clinic, University Health Network, Toronto, ON Canada

**Keywords:** Obesity, Epidemiology

## Abstract

**Objective:**

Copy number variations (CNVs) may contribute to medical conditions. However, research on the impact of individual CNVs on endocrine disease is limited. This study aimed to provide new data on obesity and metabolic syndrome (MetS) in adults with microdeletion 22q11.2, the pathogenic CNV associated with 22q11.2 deletion syndrome.

**Methods:**

We examined prevalence rates of obesity and MetS in 103 adults with a typical 22q11.2 deletion (45.2% male, at median age 30.0 (range 17–71) years) and compared these rates with population-based data. Generalized obesity was defined by a body mass index (BMI) ≥ 30 kg/m^2^, abdominal obesity by a waist circumference (WC) of ≥102 cm in males and ≥88 cm in females, and MetS by standard Joint Interim Statement criteria. General linear models were used to examine the independent associations of age, sex, congenital heart defect, smoking, and antipsychotic use with BMI, WC, and the presence of MetS.

**Results:**

Prevalence rates of generalized obesity (32.0%), abdominal obesity (51.5%), and MetS (33.0%) were significantly higher compared to a population-based cohort (15.7% (*P* < 0.0001), 36.1% (*P* = 0.002), and 15.2% (*P* < 0.0001), respectively). In antipsychotic naïve subjects, significant correlations were observed between age and BMI (*r* = 0.54, *P* < 0.001), and age and WC (*r* = 0.60, *P* < 0.001). These correlations were not present in individuals taking antipsychotic medication. The models predicting BMI (*F*(5, 97) = 3.083, *R*^2^ = 0.137, *P* = 0.01) and WC (*F*(5, 92) = 5.985, *R*^2^ = 0.245, *P* < 0.001) were significant. Only age was individually predictive of outcomes (*P* < 0.05 and *P* < 0.001). The model predicting MetS was also significant (*P* < 0.001), with higher age being the only factor associated with MetS (OR = 1.07, 95% CI = 1.03–1.12, *P* < 0.001).

**Conclusions:**

Generalized and abdominal obesity, as well as MetS, appear to be common in adults with 22q11.2 deletion syndrome, emphasizing the importance of careful monitoring from a young age. These findings contribute to the limited knowledge about the association between pathogenic CNVs, obesity, and MetS.

## Introduction

Cardiovascular and cardiometabolic health are determined by the complex interplay of genetic, socioeconomic, and environmental factors [1]. For example, genetically predisposed obesity, one of the risk factors for cardiometabolic disease, includes monogenic, polygenic and syndromic causes [1]. With advances in genomic medicine, pathogenic copy number variations (CNVs) are increasingly recognized as important contributors to genetic disease risk [2–5]. One example is the recurrent 22q11.2 deletion, which is associated with the most common human microdeletion syndrome [6], 22q11.2 deletion syndrome (22q11.2DS; OMIM #192430, #188400). Two previous single-center cohort studies in adults with 22q11.2DS reported high prevalence rates of generalized obesity (43.5%; 90/202 Canadians and 38.5%; 20/52 Belgian, respectively), based on body mass index (BMI) [7, 8], the most widely used and a quick measure of obesity [9]. In the Canadian study, age and antipsychotic medication use were associated with higher obesity levels [7, 10]. However, in the smaller Belgian study, the proportion of patients on antipsychotic medication did not differ between those with and without obesity [8]. Additionally, two other studies from the same Canadian adult 22q11.2 cohort suggested that 22q11.2DS is a significant independent risk factor for type 2 diabetes [11], and hypertriglyceridemia [12]. Nevertheless, even in the most studied CNV, the 22q11.2 deletion [2], research on cardiovascular and cardiometabolic health remains limited and constrained by modest sample sizes.

Irrespective of the 22q11.2 deletion, there is a broad interindividual variation in BMI, intra-abdominal fat tissue, and obesity-related complications and morbidity [13]. Waist circumference (WC), an indicator of visceral adipose tissue, is considered a better independent risk marker of cardiovascular and cardiometabolic morbidity and mortality than BMI [14]. WC is also a component in the definition of metabolic syndrome (MetS), a clustering of risk factors for developing cardiometabolic disease. Specifically, patients with MetS have a 2-fold increased risk to develop cardiovascular diseases, and a 5-fold increased risk of developing type 2 diabetes in the next 5–10 years compared to the general population [15].

In this cohort study, we aimed to validate the previously reported findings on generalized obesity in a Dutch sample of 103 adults with 22q11.2DS [7, 8], while extending them with data on abdominal obesity [14], and MetS [15]. We hypothesized high rates of obesity and MetS in adults with 22q11.2DS, and age and history of antipsychotic medication use to be contributing factors [7, 10].

## Methods

Prevalence rates and related factors of generalized and abdominal obesity, as well as MetS were examined in an outpatient sample of adults (aged ≥17 years) with 22q11.2DS, confirmed to have a typical 22q11.2 deletion (i.e., overlapping the LCR22A to LCR22B region) by standard methods [6].

### Ethics approval and consent to participate

A waiver for formal approval under the Medical Research Involving Human Subjects Act (WMO) was obtained from the Institutional Review Board of Amsterdam UMC, the Netherlands (#W20_098). Written informed consent to use clinical data was obtained from all participants and/or their substitute decision-makers. All data were collected and analyzed according to the EU General Data Protection Regulation and the Dutch General Regulation Data Protection (Uitvoeringswet AVG).

### Subjects

The study sample comprised 103 participants seen at a specialized 22q11.2 clinic at ‘s Heeren Loo, the Netherlands, between January 2019 and February 2024. Potential participants were included if they had adult data (collected at ≥17 years of age) on height and weight. Participants were referred from three main sources: family medicine (*n* = 38, 35.9%), intellectual disability medicine (*n* = 34, 33.0%), and pediatrics (*n* = 16, 15.5%). The most common reasons for referral were periodic 22q11.2DS-related assessments and health monitoring (*n* = 63, 61.2%; including transition to adult care), and psychiatric symptoms and/or psychosocial problems (*n* = 23, 22.3%). Two individuals (1.9%) were referred primarily for weight-/ cardiometabolic-related issues.

### Data collection

We used available lifetime medical records for all adults to collect information on age, sex, ethnicity, the presence and complexity of a congenital heart defect (CHD) [16], the presence and severity of intellectual disability based on standard criteria [17], and the history of any endocrinopathies and/or cardiometabolic disease, smoking, and medication use. Height, weight, WC, and blood pressure measurements were obtained as part of a standard assessment at the clinic. In the rare instances where these data were not collected at the 22q11.2 clinic, we used the latest measurements documented in medical records, which were verified as physician-obtained. We used the most recent available blood test results.

### Generalized and abdominal obesity

Generalized obesity was defined as a BMI ≥ 30 kg/m^2^ and categorized into class 1 (BMI ≥ 30, <35 kg/m^2^), class 2 (BMI ≥ 35, <40 kg/m^2^) and class 3 (BMI ≥ 40 kg/m^2^) [18]. BMI was calculated as weight in kilograms divided by height in meters squared. For one individual who had undergone a gastric bypass prior to the first visit to the 22q11.2DS outpatient clinic, the weight and BMI recorded before surgery were used in the analyses. Abdominal obesity was defined as a WC of ≥102 cm in males and ≥88 cm in females [14]. WC was measured by a physician at the approximate midpoint between the lower margin of the last palpable rib and the top of the iliac crest, at the level of the navel, at the end of a normal expiration. We used the maximum available adult BMI and WC measurements for analyses.

### Metabolic syndrome

MetS was defined as having 3 to 5 of the following criteria, according to the Joint Interim Statement (JIS) criteria: (1) elevated WC, (2) elevated blood pressure (systolic ≥130 mmHg and/or diastolic ≥85 mmHg) and/or antihypertensive drug use with a history of hypertension, (3) elevated fasting glucose ( ≥ 5.6 mmol/l) and/or on glucose-lowering drugs, (4) elevated triglycerides ( ≥ 1.7 mmol/L) and/or on triglyceride-lowering drug use, (5) reduced HDL cholesterol (HDL-C; <1.0 mmol/L in males, <1.3 mmol/L in females) and/or on cholesterol-lowering drug use [15]. For those with no fasting glucose data available, we considered non-fasting glucose levels ≥11.1 mmol/l, the threshold for diagnosing diabetes [19], elevated. Conservatively, in those with missing WC data (*n* = 5), we assumed WC only to be elevated in a female with BMI 41.5 kg/m^2^, and not in the others with BMI values ranging from 19.6–31.1 kg/m^2^. For a few patients, none of whom used cholesterol or glucose lowering medication, no blood levels for glucose (*n* = 3), triglycerides (*n* = 7), and HDL-C (*n* = 7) were available.

### Comparison with prevalence rates of obesity and metabolic syndrome in the general population

To compare prevalence rates of obesity and MetS in the 22q11.2DS cohort with those in the general population, we used published data from Lifelines, a large Dutch population-based adult (≥18 years, mean age ~45 years) cohort of over 70 000 participants (~60% female) [20, 21]. In that cohort, study participants were recruited between 2006 and 2013, blood pressure and anthropometric measurements (height, weight, WC) were conducted by a nurse, and blood was collected in the fasting state for measurements of glucose, cholesterol, and triglycerides. The definitions for generalized obesity and increased WC were the same as those applied in the current study. MetS was diagnosed based on the revised National Cholesterol Education Program Adult Treatment Panel III (NCEP-ATPIII) criteria with age-adjusted thresholds for blood pressure [20, 22]. At least 3 of the 5 criteria needed to be present to diagnose MetS: (1) elevated WC, (2) elevated blood pressure (systolic mmHg ≥140 and/or diastolic ≥90 mmHg for those aged <60 years, and systolic ≥150 mmHg and/or diastolic ≥90 mmHg for those aged ≥60 years), (3) elevated fasting glucose (≥5.6 mmol/l) and/or on glucose-lowering drugs, elevated triglycerides (≥1.7 mmol/L) and/or on triglyceride-lowering drug use, reduced HDL-C (<1.03 mmol/L in males and <1.30 mmol/L in females) and/or on cholesterol-lowering drug use.

### Statistical analyses

We calculated prevalence rates with their 95% confidence intervals (95% CI) for generalized and abdominal obesity, and MetS, using the normal distribution approximation (Wald interval) formula; ${CI}={\rm{\rho }}\pm 1.96* \sqrt{\frac{\rho (1-\rho )}{n}}$. We used Fisher’s exact tests for comparing proportions between both sexes and a Mann–Whitney *U* test for comparing ages between males and females, given the asymmetric age distribution. Independent samples *t*-tests were used to compare mean BMI and WC values between males and females, and Pearson correlation coefficients were used to investigate the relationship between, age, BMI, and WC values. Chi-square tests were used to compare prevalence rates of obesity and Mets in adults with 22q11.2DS and those in the general population. General linear models were used to examine the independent association of age, sex, CHD of any severity, lifetime history of smoking, and lifetime history of antipsychotic medication use with BMI, WC, and the presence of MetS. All analyses were two-tailed, with statistical significance defined as *P* < 0.05, using IBM SPSS software (Statistics 25; SPSS, Inc., Chicago, IL).

## Results

### Descriptives

Demographic and clinical characteristics of the study participants are presented in Table 1. One hundred three adults (47 males, 45.6%) with 22q11.2DS were included in the study at a median age of 30.0 (range 17–71) years. There was no difference in age between males (median age 30.0 (range 17–71) years) and females (median age 32.0 (range 17–69) years; *P* = 0.87). The mean adult height and weight were 1.74 (SD = 8.5) m and 79.8 (SD = 21.9) kg for males and 1.64 (SD = 7.9) m and 73.9 (SD = 19.1) kg for females, respectively.

**Table 1:** Demographics and clinical characteristics of 103 adults with 22q11.2 deletion syndrome.

	Total study sample *n* = 103		Males *n* = 47		Females *n* = 56		Statistics
	*n*	%	*n*	%	*n*	%	*P*^a^
Ethnicity, European	91	88.3	40	85.1	51	91.1	0.37
Congenital heart defect^b^	41	39.8	23	48.9	18	32.1	0.11
Simple	15	14.6	6	12.8	9	16.1	
Moderate	20	19.4	14	29.8	6	10.7	
Complex	6	5.8	3	6.4	3	5.4	
Intellectual disability	78	75.7	38	80.9	40	71.4	0.36
Mild	53	51.5	23	48.9	30	53.6	
Moderate	23	22.3	13	27.7	10	17.9	
Severe	2	1.9	2	4.3	0	0	
Smoking	22	21.4	12	25.5	10	17.9	0.47
Antipsychotic medication use	40	38.8	22	46.8	18	32.1	0.16

^a^Fishers’ exact tests were used for comparisons between males and females.

^b^Cardiovascular anomalies were graded by structural complexity.

### Obesity and metabolic syndrome

Table 2 shows results for prevalence rates of obesity and MetS. Generalized obesity, abdominal obesity and MetS were present in 33 (32.0%), 53 (51.5%), and 34 (33.0%) of the study participants, respectively. The mean BMI was not significantly different between males (26.0 kg/m^2^, SD = 6.1) and females (27.4 kg/m^2^, SD = 6.6, *P* = 0.29). Also, mean WC was not different between males (96.7 cm, SD = 18.3) and females (92.8 cm, SD = 17.1, *P* = 0.27). Mean WC values exceeded criteria for abdominal obesity in females (≥88 cm), but not in males (≥102 cm).

**Table 2:** Prevalence rates of obesity and metabolic syndrome in 103 adults with 22q11.2 deletion syndrome.

	Total study sample *n* = 103				Males *n* = 47				Females *n* = 56				Statistics
	*n*	%	95% CI		*n*	%	95% CI		*n*	%	95% CI		*P*^a^
Generalized obesity (BMI ≥ 30 kg/m^2^)	33	32.0	23.0	41.1	16	34.0	20.5	47.6	17	30.4	18.3	42.4	0.83
Class 1 (BMI 30 to <35 kg/m^2^)	23	22.3	14.3	30.4	13	27.7	14.9	40.5	10	17.9	7.8	27.9	
Class 2 (BMI 35 to <40 kg/m^2^)	6	5.8	1.3	10.4	2	4.3	0	10.0	4	7.1	0.4	13.9	
Class 3 (BMI > 40 kg/m^2^)	4	3.9	0.2	7.6	1	2.1	0	6.3	3	5.4	0	11.3	
Abdominal obesity (elevated WC)^b^	53	51.5	41.8	61.1	20	42.6	28.4	56.7	33	58.9	46.0	71.8	0.12
Metabolic syndrome^c^	34	33.0	23.9	42.1	14	29.8	16.7	42.9	20	35.7	23.2	48.3	0.54

*BMI* body mass index, *WC* waist circumference.

^a^Fisher’s exact tests were used for comparisons between males and females.

^b^Abdominal obesity was defined as a WC of ≥102 cm in males and ≥88 cm in females.

^c^Metabolic syndrome according to the Joint Interim Statement criteria (See Methods for details).

Compared to the Dutch population-based group (Lifelines), a significantly greater proportion of adults with 22q11.2DS had generalized obesity (32.0% (95% CI = 23.0–41.1%) vs 15.7% (95% CI = 15.5–15.9%), χ^2^ = 20.76, df = 1, *P* < 0.0001), abdominal obesity (51.5% (95% CI = 41.8–61.1%) vs 36.1% (95% CI = 35.8–36.4%), χ^2^ = 10.55, df = 1, *P* = 0.002), and MetS (33.0% (95% CI = 23.9–42.1%) vs 15.2% (95% CI, 15.0–15.5%), χ^2^ = 25.20, df = 1, *P* < 0.0001) [20, 21].

### Relationships between age, BMI, and WC

**Fig. 1:**
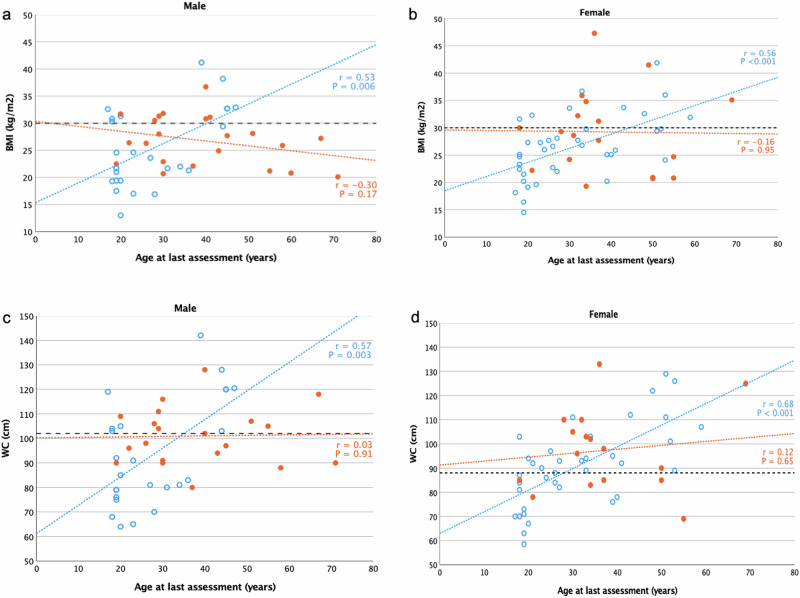
Scatterplots of the relationship between age and BMI, and age and WC. Blue open dot symbols indicate adults without, and orange filled dot symbols those with, history of antipsychotic medication use. Pearson correlation coefficients are shown to the right of the plots. **a**, **b** The horizontal dashed lines indicate the threshold for generalized obesity (BMI ≥ 30 kg/m^2^). **c**, **d** The horizontal dashed lines indicate the threshold for abdominal obesity (WC ≥ 102 cm in males and ≥88 cm in females). Five adults (two males) had no WC data. *BMI* body mass index, *WC* waist circumference.

Moderate to strong positive correlations between age and BMI (*r* = 0.54, *P* < 0.001), and age and WC (*r* = 0.60, *P* < 0.001), were found in antipsychotic naïve subjects. No statistically significant interactions were found between age and BMI (*r* = −0.14, *P* = 0.38), and age and WC (*r* = 0.08, *P* = 0.65), in those with antipsychotic medication. There was a strong positive correlation between BMI and WC values both in males (*r* = 0.93, *P* < 0.0001) and females (*r* = 0.86, *P* < 0.0001). Figure 1 shows the relationship at the individual level between age and BMI (Fig. 1a, b), and age and WC (Fig. 1c, d), divided by sex.

### Factors related to obesity and metabolic syndrome

Table 3 summarizes the results of the regression models that examined the independent associations between demographic and clinical factors, and BMI, WC, and the presence of MetS. The multiple regression models predicting BMI (*F*(5, 97) = 3.083, *R*^2^ = 0.137, *P* = 0.01) and WC (*F*(5, 92) = 5.985, *R*^2^ = 0.245, *P* < 0.001) were statistically significant, explaining 11.9% and 21.6% of the variance, respectively. Only age had a significant independent association with BMI and WC, meaning that higher age was associated with higher BMI (*P* < 0.05) and WC (*P* = 0.001). The logistic regression model predicting the presence of MetS was also significant (likelihood ratio test: χ2 = 3.1, df = 8, *P* < 0.001). Only higher age was associated with the presence of MetS (OR = 1.07, 95% CI = 1.03–1.12, *P* = 0.001).

**Table 3:** Contributing factors to BMI, waist circumference and metabolic syndrome in 103 adults with 22q11.2 deletion syndrome.

	BMI					WC^a^					MetS			
	*B*	*SE B*	β	*t*	*P*^b^	*B*	*SE B*	β	*t*	*P*^b^	OR	95% CI		*P*^b^
Age at last assessment	0.10	0.05	0.21	2.00	**<0.05**	0.51	0.13	0.39	3.92	**<0.001**	1.07	1.03	1.12	**<0.001**
Female sex	1.07	1.25	0.08	0.86	0.39	−4.71	3.31	−0.13	−1.42	0.16	1.44	0.55	3.78	0. 46
Congenital heart disease	−2.44	1.30	−0.19	−1.88	0.06	−5.58	3.44	−0.16	−1.62	0.11	1.19	0.44	3.24	0.74
Smoking	1.62	1.54	0.10	1.05	0.30	3.62	4.09	0.08	0.89	0. 38	1.80	0.62	5.28	0.28
Antipsychotic medication	0.25	1.36	0.02	0. 81	0.86	0.02	3.61	0.00	0.01	1.00	0.72	0.26	2.01	0.53

Standard multiple regression analyses were performed to examine the independent associations between demographic and clinical factors, and BMI and WC. In addition, a logistic regression analysis was performed to identify the variables predictive of MetS.

*BMI* body mass index, *WC* waist circumference, *MetS* metabolic syndrome, *B* unstandardized coefficient, *SE* standard error, *ß* standardized coefficient, *OR* odds ratio, *CI* confidence interval.

^a^*n* = 98; five patients had no data for WC.

^b^Bold font indicates statistical significance.

## Discussion

The results of this study suggest that the predisposition to develop obesity in adults with 22q11.2DS is twice as high as in the Dutch general population [20, 21], with 32.0% of the study sample having generalized obesity. Adding to the knowledge on generalized obesity, these results suggest that adults with 22q11.2DS have a marked increased risk of abdominal obesity. Approximately half of the study sample (52%) had abdominal obesity, versus 36% in the Dutch population-based cohort [21]. This is important because, although there is a strong relationship between generalized and abdominal obesity, abdominal obesity is a cardiovascular risk factor independent of BMI [13]. Moreover, we report for the first time, a markedly increased prevalence of MetS in 22q11.2DS compared to rates reported in the general population [20]. Taken together, the findings of this study contribute to the growing evidence that cardiometabolic disturbances are common in adults with 22q11.2DS [7, 8, 11, 12, 23], which can subsequently lead to cardiovascular disease and other health risks.

The findings are in line with a study that reported an increased prevalence of obesity in a cohort of Canadian adults with 22q11.2DS, showing an odds ratio of 2.3 [7]. However, obesity prevalence was higher in the somewhat younger Canadian cohort (43.5% at a median age of 27 years). In addition to environmental and lifestyle differences, considerable variation in BMI due to genetic background and body composition should also be considered [9]. Compared to Canada, the Netherlands has a genetically more homogeneous population. Indeed, fewer participants in the Dutch cohort were of non-European ethnicity compared to the Canadian cohort [24]. On average, Dutch males were 5 cm, and females 7 cm taller, than Canadians. Obesity prevalence among Belgians, who are geographically and genetically closely related to the Dutch, was 38.5% (at a median age of 30 years).

Unexpectedly, the lifetime history of antipsychotic medication use was not a significant individual contributing factor to the degree of obesity or the presence of MetS. While the Canadian study involving 202 adults with 22q11.2DS showed an association between antipsychotic medication use and higher BMI class [7], the Belgian study of 52 adults did not find a difference in antipsychotic medication use between those with or without obesity and those without [8]. It may be speculated that the effect of antipsychotic medication use on the development of obesity and metabolic syndrome [10], both of which are typically multifactorial diseases, may be overshadowed by other potential contributing factors. These include the effects of the 22q11.2 deletion itself, the use of other medications with potential weight-inducing side effects, and environmental factors such as socioeconomic situation, stress, physical activity, and diet [1, 7]. It is also possible that some patients who were prescribed antipsychotic medication had already adopted healthier habits, positively affecting obesity and MetS. The lack of a positive association between age and BMI and WC in antipsychotic naïve patients, compared to those on antipsychotic medication, may support this.

Studies exploring cardiovascular, cardiometabolic, and other medical consequences of pathogenic CNVs are still in their infancy [2]. Underlying causes include both biological and environmental factors, the latter involving an unhealthy lifestyle that is more common among individuals with pathogenic CNVs, often due to coexisting learning difficulties. Although specific genes and/or biological mechanisms are believed to be responsible for the increased risk of developing cardiometabolic conditions in other CNVs (e.g. [25, 26]), no gene or mechanism is known to increase the propensity for obesity and/or MetS in 22q11.2DS. Among the approximately 50 protein-coding genes [6], the catechol-*O*-methyltransferase (COMT) gene, which is involved in the degradation of catecholamines, including dopamine, and six genes involved in mitochondrial functioning [27, 28], may be considered candidate genes. Disruptions in the dopamine system and mitochondrial functioning have been implicated both in 22q11.2DS, and obesity and MetS [10, 29–34]. Moreover, as hypothesized for other conditions [12, 35], it is also possible that the presence of the 22q11.2 deletion may reduce the degree of background polygenic risk required for the development of metabolic disturbances.

### Implications and future directions

The findings of this study underscore the need to promote healthy lifestyle behaviors, including exercise and a healthy diet, and to conduct periodic cardiovascular risk assessments for all adults with 22q11.2DS [23]. However, it should be noted that no studies have yet investigated the feasibility and effectiveness of such interventions in 22q11.2DS. Although the lifetime risks and outcome of cardiovascular disease in 22q11.2DS have not yet been fully reported, cardiovascular disease, including more severe forms of CHDs, appears to be a major factor contributing to reduced life expectancy in 22q11.2DS [36]. Longitudinal prospective studies in 22q11.2DS are needed to gain a better understanding of the factors involved in the development of obesity and MetS, and to evaluate the effects of interventions aimed at reducing obesity, MetS, and other obesity-related diseases. This is particularly relevant in the rapidly evolving era of anti-obesity medications [37], which are effective for common obesity and have recently shown promising effects in monogenic and syndromic obesity [38]. Further research on the pathophysiology of obesity in individuals with 22q11.2DS is needed to identify the causative and sustaining factors. The potential role of hyperphagia in some individuals, as well as the effect of different types, doses, or duration of antipsychotic medication should be considered in this regard. Future studies that include data on parental obesity, other genetic risk factors (in addition to the 22q11.2 deletion), and social risk factors may further clarify the biological impact of the 22q11.2 deletion on the development of obesity and MetS.

### Strengths and limitations

The current study has several strengths, including the fact that all study participants underwent standardized clinical assessments, which involved careful history-taking, collection of collateral information, and measurement of height, weight, and WC. To our knowledge, this study is the first to evaluate WC and MetS in 22q11.2DS and one of the first systematic studies exploring the associations between pathogenic CNVs and cardiovascular risk factors. Ascertainment was unbiased with regard to recruiting adults with weight-/ cardiometabolic-related issues, though a relatively large number of patients were referred through intellectual disability medicine. With respect to limitations, we acknowledge the absence of a head-to-head comparison group (e.g., a general population sample, individuals with intellectual disabilities without 22q11.2DS, or individuals with a 16p11.2 deletion, another pathogenic CNV associated with high rates of obesity) [4, 5]. Such a comparison group could have helped better account for age and sex distribution, compare differences in penetrance and severity of obesity and determine to what extent the predisposition to obesity and MetS is directly related to the 22q11.2 deletion or has a more indirect relationship. Comorbid medical conditions, relatively low socioeconomic status, physical inactivity, stress, and unhealthy diet [1, 39, 40], should be considered as potential contributing factors in this respect. Additionally, we did not have direct access to Lifelines data and therefore used aggregate data from previous studies instead [20, 21]. Thus, it is difficult to estimate the extent to which age distribution has influenced the results. It is also unknown how representative the data are to the entire population of individuals with 22q11.2DS. While this was a single-center cohort, patients were seen from across the country as the clinic is a National 22q11.2 expert center. Nevertheless, more studies in 22q11.2DS are needed, especially in lower-income countries. In this clinically-based study, fasting glucose data were not available for all participants. Therefore, we slightly deviated from standard the JIS criteria for MetS and used non-fasting glucose for individuals without fasting glucose data (see Methods). However, since none of those participants had a history of (pre)diabetes, or elevated non-fasting glucose levels (≥11.1 mmol/L), it is unlikely that this deviation affected the overall results.

In conclusion, this study is the first to suggest high prevalence rates of abdominal obesity and MetS in adults with 22q11.2DS. The findings contribute to the limited body of knowledge on the association between pathogenic CNVs, obesity, and MetS.

## Data Availability

The data are not publicly available due to ethical restrictions and privacy concerns. Any data requests can be directed to the corresponding author.
